# Cultures of care? Animals and science in Britain

**DOI:** 10.1111/1468-4446.12706

**Published:** 2019-11-04

**Authors:** Carrie Friese, Nathalie Nuyts, Juan Pablo Pardo‐Guerra

**Affiliations:** ^1^ LSE, Sociology; ^2^ Civics Consultancy; ^3^ Sociology Department University of California San Diego

**Keywords:** Care, civic epistemology, humanitarianism, laboratory animals, national culture, science

## Abstract

It is becoming increasingly common to hear life scientists say that high quality life science research relies upon high quality laboratory animal care. However, the idea that animal care is a crucial part of scientific knowledge production is at odds with previous social science and historical scholarship regarding laboratory animals. How are we to understand this discrepancy? To begin to address this question, this paper seeks to disentangle the values of scientists in identifying animal care as important to the production of high quality scientific research. To do this, we conducted a survey of scientists working in the United Kingdom who use animals in their research. The survey found that being British is associated with thinking that animal care is a crucial part of conducting high quality science. To understand this finding, we draw upon the concept of ‘civic epistemologies’ (Jasanoff 2005; Prainsack 2006) and argue that ‘animals’ and ‘care’ in Britain may converge in taken‐for‐granted assumptions about what constitutes good scientific knowledge. These ideas travel through things like state regulations or the editorial policies of science journals, but do not necessarily carry the embodied civic epistemology of ‘animals’ and ‘science’ from which such modes of regulating laboratory animal welfare comes.

It is becoming increasingly common to hear life scientists in Britain say that scientific research relies upon high quality laboratory animal care (Davies, 2010; Friese, 2013; Hurst and West, 2010) . The idea here is that ‘happy animals’ make ‘good science’ in that they introduce fewer confounding variables into research (Poole, 1997). The introduction of the widely cited ‘Animal Research: Reporting of *In Vivo* Experiments’ (ARRIVE) guidelines, which requires authors to report animal husbandry practices in their scientific journal articles, attests to this increasing focus on animal care within science at the institutional level (Kilkenny et al., 2010). And there is a growing discourse positing the need to create a ‘culture of care’ in laboratories and animal houses in order to ensure the well‐being of animals used in research, which seeks to make change at the organizational level while exceeding animal welfare (e.g., regulatory) requirements (Davies, Greenhough, Hobson‐West and Kirk 2018; Klein and Bayne, 2007).

However, the idea that animal care is a crucial part of scientific knowledge production is at odds with previous social science and historical scholarship regarding laboratory animals. Earlier research has shown that scientists learn to distance themselves from laboratory animals as part of their education, so that they understand animals as ‘tools’ rather than sentient creatures (Birke, Arluke and Michael 2007: 11, 14). While animal care work has been professionalized since the middle of the twentieth century (Druglitro, 2017; Kirk, 2008, 2010, 2012, 2014), it has nonetheless been marginalized relative to science per se. This is evidenced by the systematic erasure of animal husbandry practices from scientific journal articles (Birke et al., 2007; Holmberg, 2011; Lederer, 1992; Lynch, 1989), and the perceived need for the ARRIVE (Animal Research: Reporting of *In Vivo* Experiments) guidelines. In this context animal husbandry (i.e., the work involved in feeding, housing, handling and reproducing laboratory animals) has been thought of as an extra‐scientific concern that animal technicians and veterinarians are responsible for, as opposed to scientists themselves (Birke et al., 2007; Greenhough and Roe, 2011; Holmberg, 2011). Indeed, previous research has indicated that scientists do not see animal care as part of science (Lynch, 1989), and notions of objectivity have been used to support this (Birke et al., 2007).

How are we to understand this discrepancy? To begin to address this question, this paper seeks to disentangle the values of scientists in identifying animal care as important to the production of high quality scientific research. To do this, we conducted a survey of scientists working in the United Kingdom who use animals in their research. The survey examined scientists’ attitudes about the importance of animal care for different aspects of scientific work, alongside various demographic, attitudinal and work‐related questions. We defined animal care in the survey as ‘the state of the animal across its lifetime, and the treatment it receives. This includes its veterinary treatment, housing, nutrition, and stimulation as well as handling within the animal house and as part of experimental research.’ Here we sought to define animal care as incorporating but also exceeding animal husbandry by raising the affective dimensions of caring relations in relationship to states of being. The survey indicates that being British is associated with thinking that animal care is a crucial part of conducting high quality science. Nationality was addressed in the survey with a write‐in box, and so was a self‐identified category for survey participants – one that we assume is linked to one’s passport. This paper argues that through the survey we see a more stable attitudinal object emerge, one that seems tied to concrete ideas of placeness and national identity. While there are multiple pathways to becoming ‘British’ (e.g., birth or migration), it seems to be an identity that matters in the field because it carries with it a particular sense of the importance of animals and care for scientific knowledge production.

To understand this finding, we draw upon Jasanoff’s notion of ‘civic epistemologies’ (2005), which refers to ‘the systematic practices by which a nation’s citizens come to know things in common and to apply their knowledge’ (Jasanoff, 2005). Often used to study contested areas of science and technology policy (Prainsack, 2006), this paper extends the concept to also consider more everyday attitudes of scientists regarding what counts as valid knowledge. In the process, we use Prainsack’s (2006) specific extension of civic epistemologies through Foucaultian discourse analysis to consider how ‘animals’ and ‘care’ in Britain may converge in taken‐for‐granted assumptions about what constitutes good scientific knowledge.

We suggest that ‘care’ and ‘animals’ represent a taken‐for‐granted idea, or civic epistemology, amongst British scientists. In developing the notion of ‘civic epistemologies’ through the case study of governing cloning and stem cell research in Israel, Prainsack argued that neither Jewish moral systems nor Israeli pronatalism alone could explain the permissive approach to biotechnologies in Israel. Rather, Prainsack contends that the two discourses were instead overlapping in the self‐governance of Israeli ethicists and users alike, generating a kind of common sense that is deeply embodied, internalized and taken for granted. Prainsack states that pronatalism is not and does not need to be imposed in this context, but is rather ‘a discursively created truth … being translated by individuals into their own choices and commitments’ (Prainsack, 2006). Care and animals may represent a similar kind of civic epistemology amongst British scientists, which translates into their own commitments in evaluating scientific research.

During nineteenth‐century Britain, animals provided an important reference for the development of humanitarian thought and action. As a vulnerable group existing within the social milieu, animals – alongside the poor, the mad, slaves, women, children, the colonized and foreigners (Haraway, 1989; Ritvo, 1987; Thomas, 1983) – were deemed to require protection from tyranny and abuse. Treating animals humanely increasingly signified class status in the eighteenth century, and especially across the nineteenth century. Because these ideas became particularly potent with reference to animals in Britain, ‘animals’ and ‘care’ represents a kind of embodied common sense that shapes taken‐for‐granted assumptions about what counts as good science. Building upon Prainsack’s approach to civic epistemology, we conclude the paper by juxtaposing our research findings with the historical literature that has explored how animals have indexed social concerns in Britain to elaborate upon the civic epistemology of animals and care in Britain.

## Laboratory animals in the United Kingdom

This study is exclusively focused on the United Kingdom, where animal welfare has been an area of sustained public and political concern. There has also been a significant amount of social science and historical work conducted on laboratory animals that focuses specifically on the UK. All this provides a rich resource for analysing the survey findings.

Much of the scholarly literature on laboratory animals has focused on the anti‐vivisection movement, which has been investigated from historical and sociological perspectives (Arluke, 1991; Bittel, 2005; Elston, 2006; Herzog, 1993; Jasper and Nelkin, 1992; Lederer, 1992; Rupke, 1997; Sperling, 1988). This includes extensive research on the history of laboratory animal regulations, particularly within the UK, which was the first country to centrally govern the use of animals in science (French, 1975; Kean, 1998). The 1876 Cruelty to Animals Act established the still existing requirement that scientists receive a licence from the Home Office before conducting research involving animals. This legislation was updated with the Animals (Scientific Procedures) Act (ASPA) in 1986. ASPA maintained the need for a Home Office licence, but made it a requirement to adhere to the 3Rs – replacement, reduction and refinement of animals in scientific research. The 3Rs is a concept developed by Russell and Burch (1959) in their *Principles of Humane Experimentation*, which aimed to make animal welfare concerns central to the conduct of science (Hobson‐West, 2009; Kirk, 2018). The 3Rs require that science and scientists: (1) avoid or replace using animals in research by developing alternatives models and tools; (2) use the minimum number of animals in research through a focus on research design, only using animals to truly add to existing knowledge; and (3) minimize the pain, suffering, distress and harm caused to animals as part of research (see http://www.nc3Rs.org.). Refinement is here informed by the 5 Freedoms of animals in the UK. Instituted in 1965 with a focus on agricultural animals, the 5 Freedoms states that animals living under human control need to be free to behave normally while being free from: thirst and hunger; discomfort; pain, injury and disease; and fear or distress. The 3Rs have since gone beyond British laboratory animal regulations and are the gold standard in the ethics of research involving animals in the global circulation of science (Davies et al., 2018; McLeon and Hartley, 2018; Sharp, 2019).

Much of the earlier literature on animals and science, as well as its regulation, has been organized according to the notion of a divide between anti‐vivisection groups and scientists (Birke et al., 2007). In this context recent scholarship has emphasized that it is important to better understand how scientists themselves think about and respond to the welfare of animals used in experiments (Davies, 2012a; Hobson‐West, 2012; Sharp, 2019), and to the debates over their use in research (Birke et al., 2007). Social scientists have begun to examine how scientists and other laboratory workers respond to public debates regarding animal welfare in science (Hobson‐West, 2012; Hobson‐West and Davies, 2018; Michael and Birke, 1994a, 1994b). Using qualitative interviews, Hobson‐West has shown that British scientists understand the 3Rs as part of quality scientific work (Hobson‐West, 2009: 98), through which they legitimize their use of animals (Hobson‐West, 2009, 2012). She suggests that scientists working in Britain today are not opposed to regulation of their work, but rather see it as serving a supporting function. At the same time, the scientific use of animals has shifted from being predominantly rooted in toxicology research to the current situation where genomics is more predominant (Davies, 2012b, 2013a, 2013b, 2013c; Shostak, 2007), which is reflected in our sample.

In this context, animal care in science has been explored on the one hand in terms of a division of labour and on the other hand in terms of its epistemic consequences. Lynch’s (1989) classic work has provided a basis for both sets of concerns. Based on ethnographic research conducted within a neuroscience laboratory, Lynch distinguished between the ‘naturalistic’ and the ‘analytic’ animal. The naturalistic animal was the working concern of animal technicians and veterinarians, whose everyday knowledge of animal needs and well‐being were required for scientific research. This was distinguished from the analytic animal, of working concern to scientists. The analytic animal was the tissue samples and resulting data points that resulted from the animal body. Lynch noted that death was the moment at which the naturalistic animal was transformed into an analytic animal, and so had to be carefully orchestrated (see also Svendsen and Koch, 2013). Lynch's work has served as a basis for much of the contemporary research regarding both the professionalization of animal technicians and the role of care in science.

Kirk (2008, 2010, 2012, 2014), Druglitro (2017) and Greenhough and Roe (2011, 2018) have explored the professionalization of laboratory animal science, through which the naturalistic animal has been made a subject of scientific research. They build upon and extend earlier ethnographic research that explored the relationships between animal technicians and scientists and the corresponding division of labour (Arluke, 1991; Birke et al., 2007; Michael and Birke, 1994a). Where scientists are socialized to distance themselves from the animals and to see animals as tools, animal technicians cannot engage in this kind of emotional distancing (Birke et al., 2007: 98; Sharp, 2019). Birke et al. (2007: 107) link time spent with animals to attitudes, noting that animal technicians and junior research scientists are more likely to refer to personal disquiet regarding experiments while more experienced scientists and administrators are less likely to debate animal experimentation and to emphasize medical benefits. In other words, a concern with animal care has been thought of as something that distinguishes technicians from scientists. Love and care for animals has been a crucial part of creating a science of nurturing for laboratory animals (Druglitro, 2017; Kirk, 2010, 2014).

Recent research, however, has begun to problematize the idea that scientists relegate animal care and husbandry to technicians. Here the epistemic consequences of animal care are being probed. For example, Davies (2010; 2011; 2012a; 2012b; 2013a; 2013b; 2013c) has extended Lynch's line of inquiry, mapping the knowledge practices of researchers using genetically modified mice in the context of postgenomics. Davies (2010) notes that animal husbandry and care are becoming increasingly important factors to consider in efforts to translate research from the laboratory animal in science to the human patient in medicine (e.g., translational research), as genetic determinism has been problematized. Here animal husbandry is increasingly viewed as a confounding factor in experimental science, and is experienced as a hurdle to translation (Davies, 2010, 2012b, 2013c). Holmberg (2008, 2011) has explored care work in science, focusing on the training of both animal technicians and students. She argues that students and technicians are not merely justifying their work when they discuss loving and caring for animals; care is instead a crucial part of science itself. Nelson (2013, 2015, 2018) has shown how extensive knowledge of mice and their environment is (somewhat ironically) central to the knowledge production practices of (some) behaviour geneticists. Friese (2013) has shown how a concern with animal care and well‐being motivated a change in the experimental system used in one laboratory, as poor care of animals was linked with poor data that could not be translated to clinical contexts. And Dam and Svendsen (2018) have shown how there is a growing ‘patient‐ization’ of (some) laboratory animals in response to the difficulties of translational research.

The social science research regarding laboratory animals has almost exclusively used qualitative research methods to date. As a result, while it has been noted that contemporary scientists do not relegate animal care to technicians in the manner described by earlier social scientists, evidence of social change remains bound to case studies and ethnographic sites. Indeed, there have been calls to extend the methods used in studying laboratory animals specifically – and animals in society more generally – in order to address more widespread social processes that condition animals in society (Johnson, 2015). This paper addresses both of these limitations in the current scholarship regarding laboratory animals by using quantitative research methods to address scientists' attitudes regarding the importance of animal care for producing scientific knowledge.

## Materials and methods

The data for this paper were gathered in the context of a larger project that asks if, why and how biomedical scientists in the UK understand animal care as an important part of scientific research. The first part of this study was based on a survey. The survey addressed the following topics: socio‐demographics, career and work characteristics, attitudes and beliefs regarding animal care, social networks, and general values. The survey took 15–20 minutes to complete. In introducing the section of the survey that asked how important animal care was perceived to be for different aspects of being a scientist, we defined ‘animal care in science’ as follows: ‘We understand animal care to refer to the state of the animal across its lifetime, and the treatment it receives. This includes its veterinary treatment, housing, nutrition, and stimulation as well as handling within the animal house and as part of experimental research.’

We followed a random sample procedure in selecting the respondents for a survey from a self‐constructed database of UK‐based authors who published an article on biomedical research, which used animals, between 1 January 2011 and 31 December 2014. From this database of 49,164 unique authors, we created a random sample. Taking into account possible outfall due to the mobility of researchers and missing contact information, we took a random sample of 2,000, with the aim of getting a final sample of around 1,000 scientists. For each of the 2,000 selected researchers, we checked the contact information (e‐mail and address details) manually with online information. As this was a labour‐intensive process the random sample could not be enlarged. In total, 1,164 scientists were contacted in the last week of June 2015 with a request to participate in our research by completing an online survey. To optimize the response rate, the initial email was followed up by e‐mail reminders and a paper version was distributed by post in early September 2015. The survey had a response rate of 37 per cent.

Due to the way the initial database was constructed, some of these respondents were not actively using animals in experimental research. Furthermore, some respondents did not fully complete the survey. In total we received 172 valid and completed surveys of which the data is used below. Because scientists in industry and government were under‐represented due to the sampling method, this group was additionally targeted by snowball sampling, which resulted in an additional 58 useable surveys. The snowball sample had a significantly higher percentage of women (63.8 per cent) than the random sample (41.5 per cent, *t*(227) = 2.98, *p* = 0.003), and the respondents were significantly younger (average age of 38 in the snowball sample and 43 in the random sample, *t*(219) = 3.11, *p* = .002).

The composition of the total sample was the following: 47% of our respondents were women while 53% were men. The average respondent is 42 years old (SE=11). 69% of the respondents identified as British nationals. The sample under‐represents managers, senior managers and full professors (our sample has 21.07% against an estimated 35.6% in the UK) and over‐represents lower‐managerial and research scientists (61.76% in our sample against 42.3% in the UK) as well as lower‐status positions, including PhD students and laboratory technicians (17.15% in our sample against 14.3% in the UK) (Royal Society, 2014). For privacy reasons the Home Office does not provide statistical information on gender, age or any other characteristics of license holders in the UK (i.e. individuals licensed to undertake animal research), and so we cannot judge the representativeness of our sample on these variables. We can however assess other important variables, which do indicate a fairly good representation of scientists in the UK working with animals in their research. 

First, the species the respondents used in their research are found to be well distributed and representative. In the survey the species worked with was assessed using the same lexicon as Home Office licence applications. In the survey, 92% of the respondents work with mammals, while 15% work with non‐mammals; 7% of the respondents work with non‐Home office regulated invertebrates. (But note that scientists can and do work with multiple species at the same time.) The mammals most often used by respondents in our sample are mice (73%), rats (37%) and dogs (13%). The respondents using mice are not overrepresented in our sample as the official statistics show that in 2015 in the UK the most commonly used animal was the mouse, and totalled 75% of the animals used (Home Office, 2016). 

Second, there is a good institutional distribution that is representative. Among the survey respondents, 73% work within academia, 15% within industry, 10% in research institutes; the remaining 2% work in other institutions (i.e. charity or government). According to the official Home Office statistics, academic institutions are executing the most procedures (48% of the total procedures) and hold the most project licences (78% of the total number of project licences) (Home Office, 2016). While it is difficult to derive from these official statistics the distribution of the actual number of personal licence holders per type of institution, we do not feel that the high number of academic respondents is a misrepresentation of the population.

Finally, respondents were asked to indicate the field(s) in which they worked from a list based on the Home Office licence application forms. All 26 fields from the list are present in our sample. The smallest field – dentistry – represents only 2% of the respondents, while the largest field – molecular biology – employs 30%. Other large fields in the sample are immunology/immunity (23%), physiology (21%), genetics (19%), and cancer research (19%). The Home Office (2016) reported in 2014 that oncology, immunity and nervous system research were the fields using the largest percentage of laboratory animals; these are all areas of medical research that rely heavily upon molecular biology. The fields covered and the proportion amongst survey respondents appears to therefore be representative.

The descriptive statistics regarding the composition of our sample shows that it is representative with regard to type of institution, research field, and species used in experiments. Although there is some bias towards lower‐status science positions, it is not disproportionate and should be expected given the nature of the survey; managers, senior managers and full professors in science are far less likely to spend time in the laboratory, working with laboratory animals. Surveys that question sensitive topics, such as the usage and care of animals in research, run however the risk of a non‐response bias because some people will feel less inclined to answer or even refuse to participate. In our sample, we run the risk of under representing the people that do not see care for animals as an essential part of research. In Table 1 we see that the attitudes of scientists regarding animal care in scientific research are rather strongly skewed towards the right side of the scale (see Table 1 for a distribution of the frequencies). The means vary from 4.38 (SE=0.80) for designing experiments to 4.54 (SE=0.72) for reproducing findings. Even though the distribution for these variables is skewed towards the opinion that care for animals is very to extremely important for research, ten to fifteen percent of the respondents did express a differing opinion about care. Nonetheless, the possibility remains that this last group is underrepresented because of their choice to not participate in the survey.

**Table 1:** Frequency distribution of recoded Likert variables

	High quality data	Reproducing findings	Designing experiments	High quality science
Important or less	9.01	9.01	15.38	11.31
Very important	28.38	26.13	29.41	28.51
Extremely important	62.61	64.86	55.20	60.18
*N*	222	222	221	221

**Table 2:** Active variables with corresponding categories grouped along type of capital

Cultural capital: 4 variables with 21 categories	
Occupational position	Manager, non‐academic scientist, PhD student, post‐doc, faculty staff, research/technical support, senior management (p), missing (p)
Location current organization	London, Oxbridge, other, missing (p)
Institution of PhD	Abroad, no information, no PhD, non‐Russell, Russell, missing (p)
Type of research	Mixed, basic, applied, missing (p)
Economic capital: 7 variables with 24 categories	
Industry funding	Yes, no, missing (p)
Government funding	Yes, no, missing (p)
Research council funding	Yes, no, missing (p)
Charity funding	Yes, no, missing (p)
3Rs funding	Not applied, Applied and received, Applied but not received, missing (p)
Budget of lab	Less than 500.000, More than 500.000, Do not know, missing (p)
Size of lab	1‐5, 6‐15, More than 15, missing (p)
Social capital: 3 variables with 15 categories	
Time with animal	Very often, Regularly, Less than once per month, Never, missing (p)
Time with technicians	Very often, Regularly, Less than once per month, Not applicable, missing (p)
Time with NACWO	Very often, Regularly, Less than once per month, Not applicable, missing (p)

Note:

14 variables, 46 active and 15 passive categories (denoted p).

We formulated four hypotheses based upon initial ethnographic research conducted by Friese. These hypotheses are:

1. Attitudes regarding animal care are correlated with position in the ‘field’ of science (Bourdieu, 1984, 1987, 2006).
2. Attitudes regarding animal care are correlated with gender.
3. Attitudes regarding animal care are correlated with age.
4. Attitudes regarding animal care are correlated with nationality.

We tested these hypotheses with two methods: multiple correspondence analysis (Hypothesis 1) and logistic regression (Hypotheses 2–4). While Bourdieu was critical of factorial research, MCA has been combined with regression in order to better understand the patterns that make up the field in other studies (Bennett et al., 2009; see also Hess 2011). Drawing on this type of research, we decided to use two different methods to assess the different hypotheses that arose in the pilot study. In the remainder of this section, we explain the methods and describe the variables used in the analyses.

### Multiple correspondence analysis

Multiple correspondence analysis (MCA) is a quantitative method often used to operationalize Bourdieu’s field theory. MCA is a relational mode of analysis that positions individuals in relation to one another based on their similarities and differences across a multi‐dimensional space. We selected variables for mapping the field of science with the goal of having a fairly even representation of the different types of capital within science: cultural, economic and social capital. We included 14 variables to make up the three types of capital (see Table 2). Cultural capital was assessed through occupational position, the location of the current organization, the institution at which the researcher received their PhD and the type of research currently conducted. Occupational position is included as an institutionalized form of cultural capital. Scientific institutions formally recognize a person's cultural capital through awarding titles, positions and esteem. The location of their organization is included as cultural capital, because depending on its geographical location organisations are expected to provide a particular setting endowed with knowledge, culture and intellectual tradition in which individuals get socialised. Universities in London along with Cambridge and Oxford performed significantly better in the last Research Excellent Framework, a national research assessment exercise performed regularly by the state within the UK. We used this category – colloquially referred to as ‘the golden triangle’ – to denote the clear cultural capital of Cambridge and Oxford but also London‐based universities like Imperial College London, University College London and Kings’ College London. We were trying to measure how the esteem of an institution translates into the ‘pedigree’ of a scientist that is distinct from social relationships per se. Economic capital referred to the financial capacity to conduct research, and was assessed through funding type, the budget of the lab and the size of the lab. Finally, social capital is operationalized through three variables that measure the relationship intensity with actors that can provide access to knowledge and information on animal care practices. Animals are included as a non‐human actor that can provide social capital benefits; as certain information, knowledge and expertise can only be gained through the human–animal interaction.

For occupational position, we made passive the category ‘senior management’ because it contained less than 5 per cent of the respondents. For all variables, we inserted the missing category in the analysis as a passive category.

To test Hypothesis 1, we projected the opinions of scientists with regard to care for animals in scientific research as supplementary variables into the field. This makes it possible to examine whether certain positions in the field relate to particular attitudes. In particular, the importance of animal care was examined with regard to four aspects of scientific work: high quality data, reproducing findings, designing experiments and high quality science. The importance of animal care to each of these aspects of scientific work was scored by the respondents on a 5‐point Likert scale ranging from ‘not at all’ (1) to ‘extremely important' (5). The responses were recoded into three categories because the frequencies for categories 1, 2, and 3 were low. These categories were combined into the category ‘important or less’. Category 4 was recoded into a second category ‘very important’ and category 5 was turned into a third category ‘extremely important’.

### Logistic regression

We built models using linear logistic regression to test Hypotheses 2, 3 and 4. All models had, as dependent variables, scientists’ opinions regarding the importance of animal care for scientific research. For the analysis, we recoded opinions to form binary variables. Categories 1 to 3 were collapsed as ‘important or less’ and categories 4 and 5 were collapsed to form ‘very or extremely important’. The independent variables of these models were three socio‐demographic variables: gender, age and nationality. For gender, respondents could tick one of three boxes: woman, man or other. (No respondent ticked the box ‘other’, and so the variable is treated as a binary variable.) Age was measured with an open‐ended, write‐in question. Nationality was measured with an open‐ended, write‐in question. All UK nationalities (i.e., British, Scottish, English, Welsh, North Irish and UK) were recoded to 1 and all other nationalities to 0.

Importantly, nationality was self‐identified and so we cannot assess the route to or meaning of British citizenship. Based on the follow up qualitative interviews, we do know that migration to the UK through education followed by citizenship is one trajectory. Hence, not everyone who defined themselves as British was born and raised within the UK.

## Survey findings

**Figure 1:**
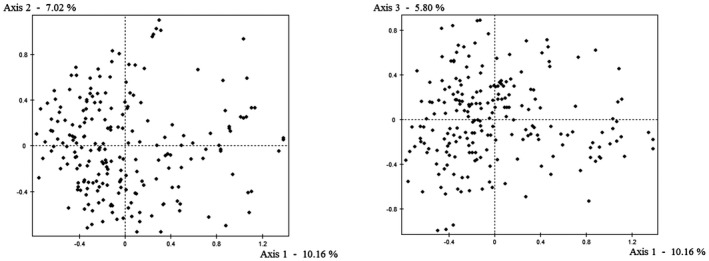
Clouds of individuals for plane 1‐2 and plane 1‐3

Hypothesis 1 states that the attitudes regarding animal care are correlated with position in the field of science. To test Hypothesis 1, we analysed the survey data using MCA as described above. The distribution of individuals was organized in a more or less circular shape, facilitating the interpretation and analysis (see Figure 1). The gap in the upper right quadrant makes empirical sense, as this is where upper management in industry science would be positioned and they are not engaging directly with animals as part of their work, and so would not fit our inclusion criteria.

Three axes were analysed to get to an accumulated eigenvalue of 82.8 per cent, which means that 82.8 per cent of the variance is explained by these three axes (LeRoux and Rouanet, 2010). Axis 1 explains 57 per cent of the variance, while axis 2 explains 18 per cent of the variance and axis 3 only explains 8 per cent of the variance (see Table 3). These three axes represent the ‘field’ of life sciences in Britain that uses animals in its research based on our sample. To interpret the axes, the contributions of the variables/categories are shown in Table 4. The contributions printed in bold exceed the minimum criterion.

**Figure 2:**
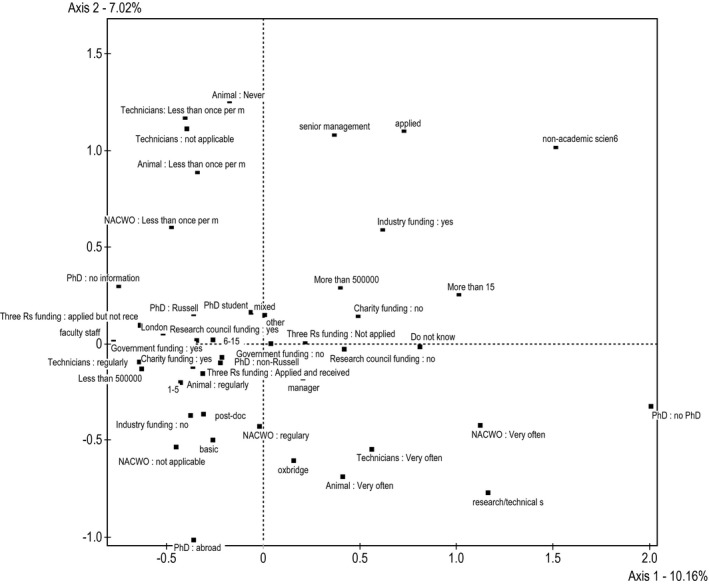
Factorial plane axis 1‐2

Axis 1 can be interpreted as distinguishing academic scientists from non‐academic scientists, positioning academics to the left and non‐academic scientists to the right. The oppositions on axis 1 are mainly from differences in cultural (35.21 per cent in total) and economic (41.53 per cent in total) capital variables. In particular, along axis 1 there are oppositions between small and large research groups, receiving industry funding or not, receiving charity funding or not, and faculty staff and non‐academic scientists. In Figure 2 (axis 1 is the horizontal axis), these oppositions are visually observable.

Axis 2 distinguishes scientists along the lines of social capital, in terms of how much they engage with animals, animal technicians and Named Animal Care and Welfare Officers (NACWOs). High levels of social capital were located on the bottom and low levels of social capital on the top of the graph (see Figure 2). Axis 2 is dominated by oppositions in social capital variables, making up 55.88 per cent of the contributions. The main oppositions on this axis are between those who interact with animals, technical support staff and the NACWO (who manages animal technicians and the animal house) less than once per month. This is on the top of the graph. Interacting with animals and technical support staff very often and with NACWOs regularly is on the bottom of the graph.

**Figure 3:**
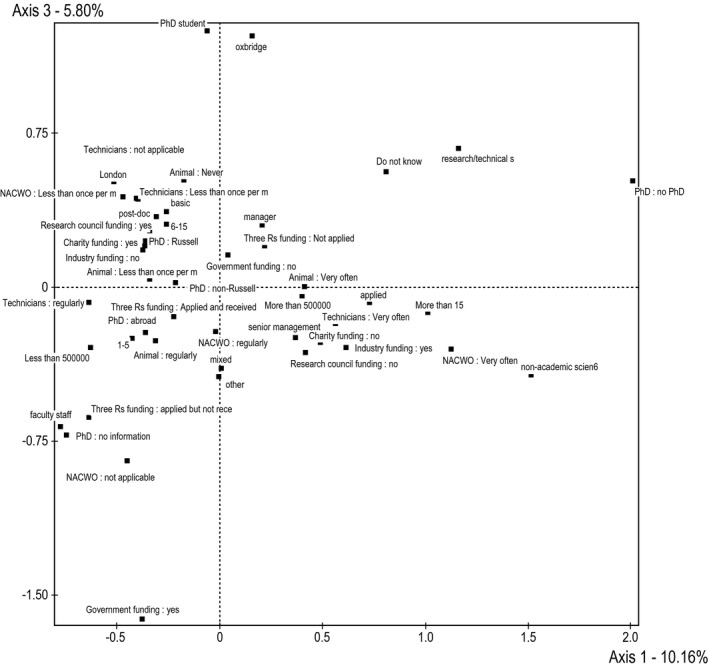
Factorial plane axis 1‐3

Finally, axis 3 distinguishes between people with a lower status in a higher esteem institution (e.g., senior technicians and PhD students in or near Oxford, Cambridge or London) from people with a higher status in a lower esteem institution (e.g., faculty outside of London and ‘Oxbridge’ with smaller budgets). Axis 3 (vertical in Figure 3) is constructed by two cultural oppositions: having one's current institution located in Cambridgeshire, Oxfordshire or London versus being in an institution with another location. Opposition on this axis is also between PhD students and support staff versus faculty staff members. This axis could be representative of the field of life science, but could also represent a bias in the sample reflecting who completed the survey.

**Figure 4:**
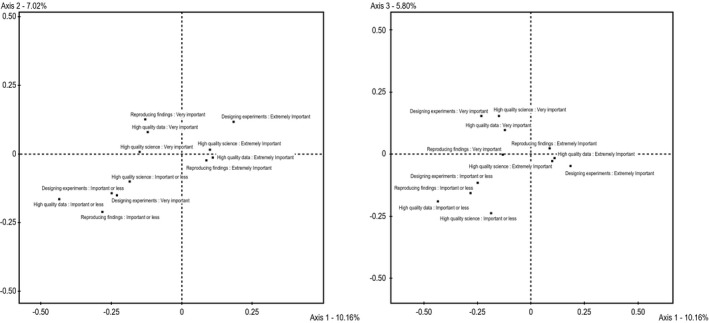
Supplementary categorical variables projected in factorial planes 1‐2 and 1‐3

Once the meaning of the axes was established, attitudes regarding the importance of animal care in scientific research were included as supplementary variables in order to test Hypothesis 1. The distance between the coordinates of the recoded Likert scales have to be at least 0.4, or at least 0.5 according to Le Roux, in order to be considered relevant. Table 5 represents the distances between the coordinates of the three categories for each attitude on each axis numerically, and Figure 4 represents this visually.

Only one of the distances reaches the more conservative distance of 0.5: the distance on the first axis between the categories of animal care is ‘important or less’ and animal care is ‘extremely important’ for high quality data are 0.544 apart. The respondents answering ‘important or less’ were concentrated on the left side of graph – indicating that scientists working in the industry were less likely to report that animal care was ‘important or less’ than academic scientists. However, respondents indicating animal care is ‘extremely important’ are not concentrated in one quadrant, but rather are present all over.

Two distances reach the less conservative distance of 0.4 on the first axis, but not the more conservative 0.5 threshold. These are both in relationship to the importance of animal care for designing experiments. Scientists working in industry were less likely to report that animal care was either ‘important’ or ‘very important’ when compared with those in academia.

Because only one value met the 0.5 criteria and only two reached the less conservative 0.4 threshold, it is concluded that there is not sufficient support for Hypothesis 1. Effects relevant in size are only found for one axis, where industry scientists place slightly greater importance on animal care. Attitudes regarding the importance of animal care for producing high quality science do not appear to be correlated with position in the scientific field. *There is no support for Hypothesis 1, which stated that attitudes regarding animal care are associated with position within the field of science*.

**Figure 5:**
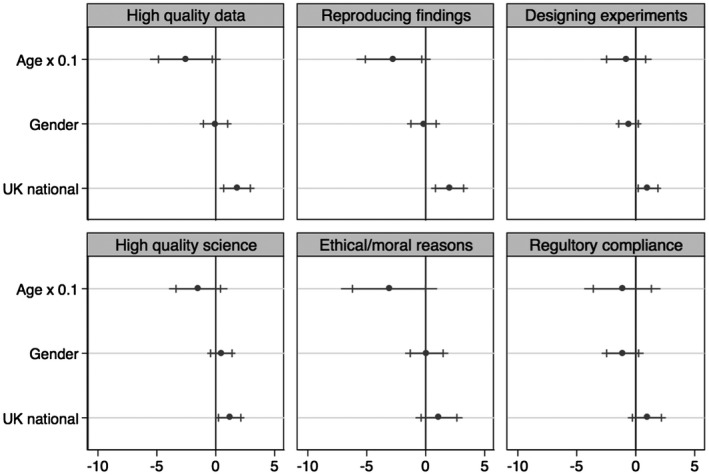
Regression coefficients for age, gender and national origin for the six predicted outcomes

Hypothesis 2 states that attitudes regarding animal care are correlated with gender.^1^ Figure 5 shows the coefficients associated from the linear logistic regression models for each category of attitudes addressing the importance of animal care for science, accounting for the demographic variables of age, nationality, and gender (for the benefit of visualization, we rescaled the variable age by a factor of 0.1, allowing for a clearer interpretation of the coefficient plot). The coefficient plot presents significance at the 99 per cent (*p* < 0.01) and 95 per cent (*p* < 0.05) levels. Gender is not significantly correlated with any of the attitudes. An examination of the close‐to‐zero coefficients suggests that this is not the result of our sample size, although future work might seek to further tease out the role of gender on attitudes about animal care in science. This may be particularly important, given patterns about the gendered division of care work in broader society and in the lab. *There is no support for Hypothesis 2, which stated that attitudes regarding animal care are gendered.*

Hypothesis 3 states that attitudes regarding animal care are partly correlated with age. Figure 5 shows a statistically significant association (*p* < 0.05) between a scientist’s age and attitudes regarding the importance of animal care for producing high quality data and reproducing research findings (see also Table A1). When age increases by 1 year, scientists are 1.06 times more likely to state the importance of animal care is ‘important or less’ rather than extremely or very important for producing high quality data and reproducing scientific findings. This is not the case for other outcomes. *There is partial support for Hypothesis 3, which states that attitudes regarding animal care differ based on age or generation.*

Finally, Hypothesis 4 stated that attitudes regarding animal care are correlated with nationality. Model 1 in Figure 5 shows that there are significant correlations between a scientist’s nationality and their reported attitudes regarding the importance of animal care for producing high quality data, reproducing scientific findings, designing experiments and producing high quality science. Being British was positively associated with thinking that animal care is extremely or very important for all aspects of scientific research measured. In particular, British scientists were six times more likely to report the importance of animal care is high for producing high quality data, almost eight times more likely to report the importance of animal care is high for reproducing scientific findings, almost three times more likely to report the importance of animal care is high for designing experiments, and three times more likely to report the importance of animal care is high for producing high quality science. *There is support for Hypothesis 4, which states that attitudes regarding animal care differ based upon nationality*.

We also tested interaction effects between the significant main results. Significant interaction effects were found between age and nationality for attitudes regarding the importance of animal care for producing high quality data (*B* = –0.13, SE = 0.06) and reproducing findings (*B* = –0.12, SE = 0.06). The effect of age on producing high quality data for non‐British scientists is 1.14 times the effect of age for British scientists. In other words, for non‐British scientists the effect of age on producing high quality data diminishes with 0.14 each year, while for British scientists this is only 0.02. Similarly, the effect of age on reproducing findings for non‐British scientists is 1.12 times the effect of age for British scientists. In other words, for non‐British scientists working in the UK the effect of age on attitudes regarding the importance of care for producing high quality data diminishes with 0.13 each year, while for British scientists this is only 0.02. *The size of the effects of the dependent variables shows that the age effect is mostly present within the non‐British scientists.*

### Additional analyses

**Figure 6:**
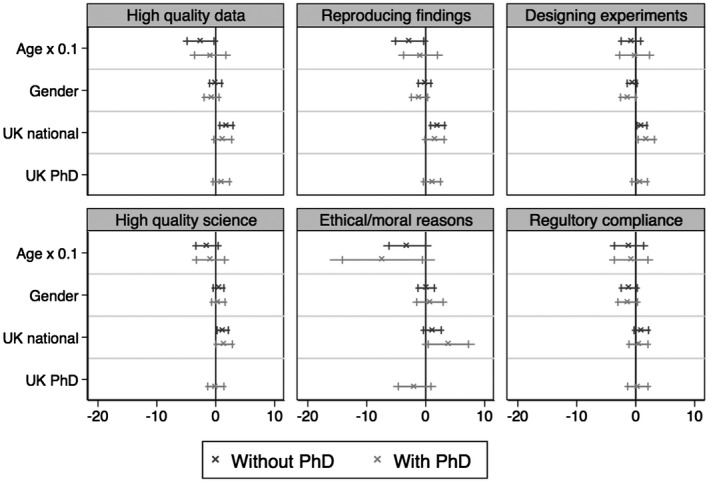
Comparison of the regression coefficients for a model 1 containing age, gender, and national origin with a model 2 that includes whether PhD was awarded in the United Kingdom

We were surprised by the strength of the correlation between nationality and attitudes regarding the importance of animal care for scientific research, and so made additional analyses in order to gain a better understanding of this finding. First, following Abbott’s (1988) theory of professionalization, we thought that this national difference may be the result of scientists' training within the UK in the context of a highly regulated state apparatus for managing animal use in science, which also mandates researchers to reduce, refine, and replace animals from their experiments. While only 3 per cent of the UK nationals had a PhD from a foreign institution, 42 per cent of the non‐British nationals received their PhD from an institution abroad. We re‐ran the logistic regression controlling for whether or not someone did their PhD abroad. Figure 6 shows that the association between attitudes and nationality remain significant for all aspects of science, but are less strong. This diminished association is in part the result of a decrease in sample size (from over 200 to 160 cases; see Table A2). *Having a UK or non‐UK PhD does not significantly relate to a scientist’s attitudes regarding the importance of animal care for research.*

Second, we wanted to assess if there was a correlation between nationality and other attitudes that were addressed in the survey. Significant correlations between nationality and these additional attitudes would imply that there is a general tendency to answer differently on Likert scales depending on nationality. The additional attitudes – the importance of animal care for regulatory compliance, for ethic/moral reasons, and for ensuring public support – were measured with the same 5‐point Likert scale as the attitudes analysed in this paper and were similarly recoded into two categories. Model 1 in Figure 5 shows the coefficient plot for the linear logistic regressions for the importance of animal care to ensure public support, for moral/ethical reasons, and for regulatory compliance. There is no association between nationality and these three attitudes. *It does not appear that there was a general tendency for British scientists to use the higher end of the Likert scale when compared with non‐British scientists* (see Table A3)*.*

We also controlled for variables that might have intervened in attitudes concerning the importance of animal care in scientific research. These included the respondent’s self‐identified location within a left‐right political spectrum, her involvement with civic organizations, and her proximity to companion animals both in the past and at the time of the survey. Controlling for these variables did not affect the results.

## Discussion and conclusion: civic epistemologies of animals and care in British science

The survey does not provide evidence to support the idea that attitudes regarding laboratory animal care are linked to position within the field of science. Birke et al. (2007: 107) had previously found that time spent with laboratory animals is linked with attitudes about the importance of animal care, such that junior research scientists are more likely to experience disquiet regarding experiments while more experienced scientists are less likely to debate animal experimentation and to emphasize medical benefits. Similarly, the scientists Friese spoke with in the ethnographic pilot study posited that those who believe animal care is a crucial part of scientific research spend more time working directly with laboratory animals. However, we did not find evidence that attitudes regarding the importance of animal care for scientific knowledge production are linked with the institutional organization of science.

The survey does, however, indicate that the idea of animal care as a crucial part of scientific research may be a distinctly British phenomenon. This scientific ideal appears to be one that younger, non‐British scientists are more receptive to when compared to older, non‐British scientists. This could be associated with the diffusion of the 3Rs since the mid‐1980s (Sharp, 2019). The data analysis thus indicates that animal care is ‘sticky’ across nationalities, rather than tied to distinct forms of scientific habituation.

We would expect that the strength of the relationship between being British and thinking that animal care is important for different dimensions of scientific work would be linked to exposure to the regulatory milieu in the UK as well as to the corresponding training that British scientists receive in the 3Rs. The way laboratory animals are regulated in the United Kingdom should, we expected, create sensibilities towards animal care. However, the fact that national differences persist even when scientists have done their PhD in the UK does not support this. Rather, attitudes regarding the importance of animal care for science appear to be located in something more general like a national culture.

This is particularly surprising given the cosmopolitan nature of science and scientific training in Britain. Just as we would expect those socialized in British science to be slightly more concerned with matters of care, we would also expect British researchers to have been exposed to diverse settings with less intense concerns for animal welfare. Indeed, what we find is variation in attitudes towards animal care based on national origin, despite the fact that scientists develop their careers in a highly standardized institutional field (scientific research) where incentive and reward structures should promote greater levels of isomorphism. Indeed, while there is a generational patterning among non‐British respondents, our results show surprisingly little variation across generations of British scientists regarding the salience of animal care for knowledge production. This seems to indicate that a relatively stable set of ideas regarding animals and/or care are shaping ideas about what counts as good science among British researchers. This provides further evidence for interpreting animal care in science as linked with a British national culture of animals and care.^2^

National culture and policy are of course highly entrenched with one another and 'co‐produced' (Jasanoff, 2005). The co‐production framework comes closest to helping us understand attitudes about the salience of animal care. Here the governance of animal welfare embodies a national culture that in turn shapes science; that science in turn shapes governance. However, because of its focus on governance, even the co‐production framework would lead us to believe that non‐British scientists who were trained and work within the UK would share similar attitudes regarding the salience of animal care given that they work within the same policy context. Rather, the findings are indicative of the type of long, embedded cultural repertoires that the notion of civic epistemology captures. What appears to be exposed here is not habituation at the laboratory bench, but earlier habits and dispositions towards animals and care that are part and parcel of British identities.

We contend that to understand this phenomenon we need to understand British discourses about animals as well as care in tandem. In making this argument, we build upon Prainsack’s (2006) development of civic epistemologies through her Foucaultian approach to discourse analysis. Prainsack (2006) argues in the case of Israeli stem cell and cloning science that regulation cannot be understood to arise from one discourse alone, either through religion or through pronatalism. Rather, the two come together in presenting ‘an Israeli solution in the full sense of the word’ (Prainsack, 2006: 196) such that any other position is inconceivable. We contend that ‘care’ and ‘animals’ converge in a similar kind of civic epistemology for British science. Neither discourse on care nor animals alone can explain the attitudes that British scientists have regarding what counts as valid knowledge.

The idea that the British have a unique love for animals is something of a national stereotype. The present‐day love for animals in the UK is generally located historically c. 1700–1900. This was the time when animals shifted from being understood as agents in their own right, and were thus punishable according to the law, to objects of human manipulation – and thus made into property (Ritvo, 1987: 1–3). Ritvo (1987) shows how this shift made it possible for the British to look upon animals sentimentally, and with emotional attachment (see also Tague, 2015). Ritvo notes that, at the beginning of the nineteenth century the English

would have been surprised to hear themselves praised for special kindness to animals. They were surrounded by evidence to the contrary but that by the end of the nineteenth century a humanitarian crusader proclaimed a ‘sentiment of tenderness for those of the sentient lower creatures … has become an element in the spiritual life so strong that the continual violation of social obligations to them is a cause of pain and revolt’. (Ritvo, 1987: 125–6)

In her study of pet keeping in eighteenth‐century England, Tague (2015) similarly finds that pet keeping was viewed as a luxury at best, and even a sin, at the start of the century but had become a sign of moral virtue by the end (see also Thomas, 1983).

Ritvo (1987) has shown that this love for animals was certainly affectively experienced, but it was always also indexing other social concern. Tague (2015) contends that the very ubiquity of animals in eighteenth‐century England made it possible for animals to figure in a full range of different human concerns. Across the range of separate and divergent animal‐related discourses in Victorian England, Ritvo thus contends there is a central theme of domination and exploitation. The naturalization of a hierarchy between human and non‐human animals within natural history paralleled and shaped the naturalization of hierarchies based on class, gender, race, nation and imperialism (Haraway, 1989; Ritvo, 1987; Tague, 2015).

Ritvo notes that animals rarely indexed the white English gentry, presumed to be at the top of the hierarchy; animals were instead negatively equated with those humans who similarly required control: the poor, the mad, slaves, women, children and foreigners (Haraway, 1989; Thomas, 1983).

Embodying the lower classes as sheep and cattle validated the authority and responsibility exercised by their social superiors. Embodying the lower classes or alien groups as dangerous wild animals emphasized the need for their masters to exercise strict discipline and to defend against depredations. (Ritvo, 1987: 6).

Meanwhile, charges of cruelty toward animals were similarly disproportionately directed at the lower classes; learning to care for animals was thus a civilizing process (Tague, 2015). But Tague (2015: 72) shows how these discourses also occupied the thoughts of those in a dominating position, who did publicly question if there were limits to their authority such that dominion becomes tyranny (see also Thomas 1983). Tague argues that the emergence of the sentimental literary genre spoke to an emerging discourse in which protecting vulnerable others through a benevolent paternalism becomes a priority. This was central to nineteenth‐century humanist thought concerned with animal welfare, child welfare and the abolition of slavery. Care and hierarchy are very much entangled here.

We suggest that care for animals in science is likely shaped by this idea regarding the need to *protect* those who are vulnerable. What we see here is thus a very specific notion of ‘care’ as protection that is linked to hierarchy, wherein those who have the power to dominate must do so with responsibility. As Thomas (1983) shows, there are traces of this ethos that can be dated back as far as the medieval period in religious doctrine particularly.

If pronatalism and religion converged to create a very Israeli solution to biotechology (Prainsack, 2006), animals and protectionism as care converge to create a very British solution to laboratory animals. In his historical analysis, Kirk (2018) notes that the 3Rs persisted despite the obscurity of the original 1959 text in part because the principle of replacement offered a fresh approach for antivivisectionists. The National Antivivisection Society, the British Union for the Abolition of Vivisection and the Scottish Society for the Prevention of Vivisection all took up the discourse of ‘alternatives’ as a means to curtail animal research, which aligned with the scientific inclusion of ‘replacement’ as one of the 3Rs. Kirk argues this was a very British way of doing politics through consensus building, enrolling anti‐vivisectionists into laboratory animal science. We add a discursive element to Kirk’s analysis, showing how a possibly more general British sensibility regarding animals alongside care as protectionism facilitated such a political practice.

This is not to say that British scientists do in fact care more about animals in practice. As Thomas (1983: 14) noted of British preoccupations with nature and rural life, whether or not this is a peculiarly British phenomenon or not, the English have for a long time liked to think it is. The same can be said of animals in science, where at least the belief of great care for research animals has become a way of thinking about that which distinguishes British science.

**Table 3:** Variance of axes, modified and cumulated rates

	Eigenvalues	Percentage	Modified rates	Cumulated modified rates
Axis 1	0.2374	10.16	56.7	56.7
Axis 2	0.1640	7.02	17.6	74.3
Axis 3	0.1356	5.80	8.5	82.8

**Table 4:** Contributions of the active variables

Occupational position	Axis 1	Axis 2	Axis 3	Industry funding	Axis 1	Axis 2	Axis 3		Axis 1	Axis 2	Axis 3
Manager	0.09	0.10	0.33	No	**2.48**	**3.57**	0.99	**Time with animal**			
non‐academic scientist	**7.76**	**5.06**	1.08	Yes	**4.18**	**5.56**	1.70	Very often	2.15	**8.60**	0.00
PhD student	0.01	0.08	**5.73**	Cumulative contribution	6.65	**9.14**	2.69	Regularly	0.71	0.26	0.90
Post‐doc	0.70	1.45	1.51					Less than once per m	0.71	**7.04**	0.01
Faculty staff	**5.41**	0.00	**7.32**	**Government funding**				Never	0.11	**7.94**	1.66
research/technical support staff	**3.82**	**2.44**	**2.22**	No	0.04	0.00	1.07	Cumulative contribution	3.68	**23.85**	2.57
Cumulative contribution	**17.78**	**9.12**	**18.20**	Yes	0.36	0.00	**11.62**				
				Cumulative contribution	0.41	0.00	**12.69**	**Time with technicians**			
**Location current organization**								Very often	**4.56**	**6.21**	0.81
London	1.49	0.02	**2.58**	**Research council funding**				Regularly	**3.08**	0.10	0.08
Other	0.00	0.16	**5.77**	No	**2.30**	0.02	**2.38**	Less than once per m	0.75	**9.16**	1.49
Oxbridge	0.10	**2.17**	**10.61**	Yes	1.83	0.01	1.89	not applicable	0.46	**5.27**	0.94
Cumulative contribution	1.60	2.36	**18.96**	Cumulative contribution	4.13	0.02	4.27	Cumulative contribution	**8.86**	**20.76**	3.31
											
**Institution of PhD**				**Charity funding**				**Time with NACWO**			
Abroad	0.49	**5.71**	0.33	No	**3.00**	0.38	1.59	Very often	**7.67**	1.57	0.97
No information	1.95	0.46	**3.21**	Yes	**2.17**	0.33	1.13	Regularly	0.00	**2.52**	0.81
No PhD	**8.51**	0.32	0.97	Cumulative contribution	5.17	0.71	2.72	Less than once per m	**2.71**	**6.48**	**4.06**
Non‐Russell	0.15	0.02	0.00					not applicable	0.34	0.70	2.11
Russell	1.42	0.37	0.93	**3Rs funding**				Cumulative contribution	**10.73**	**11.27**	**7.95**
Cumulative contribution	**12.52**	6.89	5.45	Not applied	0.93	0.00	1.33				
				Applied and received	0.27	0.08	0.21	**Cumulative contribution social capital**	23.27	55.88	13.84
**Type of research**				applied but not rece	1.94	0.06	**3.40**				
Mixed	0.00	0.36	**3.21**	Cumulative contribution	3.14	0.15	4.94				
Basic	0.92	**4.99**	**3.21**								
Applied	**2.39**	**7.92**	0.05	**Budget**							
Cumulative contribution	3.30	**13.27**	6.47	Less than 500,000	**5.62**	0.37	**2.21**				
				More than 500,000	1.04	0.79	0.03				
				Do not know	**5.28**	0.00	**4.36**				
				Cumulative contribution	**11.94**	1.16	6.60				
											
				**Size of lab**							
				1–5	1.82	0.60	1.12				
				6–15	0.77	0.00	1.86				
				More than 15	**7.50**	0.69	0.20				
				Cumulative contribution	**10.09**	1.30	3.18				
**Cumulative contribution cultural capital**	35.21	31.64	49.08	**Cumulative contribution economic capital**	41.53	12.48	37.08				

Note

: Contributions above the average contribution (i.e., 2.17 for categories and 7.14 for questions) are presented in bold*.*

**Table 5:** Distances of supplementary variable categories

High quality data	Axis 1	Axis 2	Axis 3
Important vs Very	0.314	0.244	0.286
Very vs Extremely	0.230	0.092	0.113
Important vs Extremely	0.544	0.152	0.173
Reproducing findings			
Important vs Very	0.152	0.337	0.154
Very vs Extremely	0.217	0.149	0.025
Important vs Extremely	0.368	0.188	0.180
Designing experiments			
Important vs Very	0.019	0.008	0.270
Very vs Extremely	0.415	0.267	0.201
Important vs Extremely	0.434	0.259	0.068
High quality science			
Important vs Very	0.036	0.109	0.392
Very vs Extremely	0.250	0.007	0.181
Important vs Extremely	0.285	0.116	0.211

## Supporting information


**Table A1.** Linear logistic regression models for High quality data, Reproducing findings, Designing experiments, and High quality science (standard errors are shown between brackets).
**Table A2.** Linear logistic regression models for High quality data, Reproducing findings, Designing experiments, and High quality science with control for the location of the PhD institution (standard errors are shown between brackets).
**Table A3.** Linear logistic regression models for Regulatory compliance, Ethics, and Public support (standard errors are shown between brackets).

## Notes

1 While we decided to examine the effects of age and gender on attitudes about animal care in science through logistic regressions separate from the MCA, we acknowledge that these variables can be expected to be correlated with position in the field. So as to not create an artificial separation between these factors, age and gender were also added as supplementary variables in the MCA analysis. We present the results here. A deviation between the coordinates of two modalities on an axis that is greater than 1 is regarded as ‘large’, a deviation less than 0.5 as ‘small’. For gender, the deviation between men and women is small for all axes: *d *= 0.29 for axis 1; *d* = 0.17 for axis 2 and *d* = 0.32 for axis 3. Age was recoded in four categories: <30; 31–40; 41–50; >51. On axis 1, there is a medium‐size deviation (*d* = 0.62) between the categories < 30 and > 51. All other deviations on axis 1 are small (*d* = 0.37 or lower). For axis 2, the deviations among the age categories are small with the largest deviation being 0.39. Finally, the age categories are ordered along the third axis with large deviations between the categories < 30 and > 51 (*d* = 1,36) and the categories < 30 and 41–50 (*d* = 1.15).

2 On national cultures of science, see also Yair (2019).
