# Supplementary material for: Cultures of care? Animals and science in Britain
Source: Br J Sociol. 2019 Nov 4;70(5):2042–69. doi: 10.1111/1468-4446.12706 (PMC6916317; doi:10.1111/1468-4446.12706)
Supplement: Supplementary file 1 — Table A1. Linear logistic regression models for High quality data, Reproducing findings, Designing experiments, and High quality science (standard errors are shown between brackets). Table A2. Linear logistic regression models for High quality data, Reproducing findings, Designing experiments, and High quality science with control for the location of the PhD institution (standard errors are shown between brackets). Table A3. Linear logistic regression models for Regulatory compliance, Ethics, and Public support (standard errors are shown between brackets). [file BJOS-70-2042-s001.docx]

Table A1. Linear logistic regression models for High quality data, Reproducing findings, Designing experiments, and High quality science (standard errors are shown between brackets).

|  | High quality data (N=206) | | | Reproducing finding (N=206) | | | Designing experiments (N=205) | | | High quality science (N=205) | | |
| --- | --- | --- | --- | --- | --- | --- | --- | --- | --- | --- | --- | --- |
|  |  | | OR |  | | OR |  | | OR |  | | OR |
| Constant | 3.95 | *** | 51.93 | 4.05 | *** | 57.23 | 2.07 | ** | 7.89 | 2.71 | ** | 14.97 |
|  | (1.08) | |  | (1.13) | |  | (0.79) | |  | (0.90) | |  |
| Gender^1^ | -0.02 |  |  | -0.20 |  |  | -0.64 |  |  | 0.48 |  |  |
|  | (0.53) | |  | (0.55) | |  | (0.42) | |  | (0.47) | |  |
| Age | -0.06 | * | 0.94 | -0.06 | * | 0.94 | -0.01 |  |  | -0.04 |  |  |
|  | (0.03) | |  | (0.03) | |  | (0.02) | |  | (0.02) | |  |
| Nationality^2^ | 1.86 | *** | 6.42 | 2.05 | ** | 7.77 | 1.01 | * | 2.75 | 1.22 | * | 3.39 |
|  | (0.59) | |  | (0.62) | |  | (0.43) | |  | (0.49) | |  |
| ¹ reference category is woman  ² reference category is non-UK  *** p<.001, ** p<.01, *p<.05  *Note: Odds ratios (OR) are only shown for significant estimates* | | | | | | | | | | | | |

|  | High quality data (N=160) | | | Reproducing findings (N=160) | | | Designing experiments (N=160) | | | High quality science (N=160) | | |
| --- | --- | --- | --- | --- | --- | --- | --- | --- | --- | --- | --- | --- |
|  |  | | OR |  | | OR |  | | OR |  | | OR |
| Constant | 4.04 | *** | 56.65 | 4.18 | *** | 65.12 | 2.02 | * | 7.51 | 2.72 | ** | 15.24 |
|  | (1.22) | |  | (1.31) | |  | (0.93) | |  | (1.02) | |  |
| Gender^1^ | -0.47 |  |  | -0.75 |  |  | -0.95 | + | 0.39 | 0.33 |  |  |
|  | (0.60) | |  | (0.64) | |  | (0.50) | |  | (0.53) | |  |
| Age | -0.04 |  |  | -0.04 |  |  | -0.00 |  |  | -0.03 |  |  |
|  | (0.03) | |  | (0.03) | |  | (0.02) | |  | (0.03) | |  |
| Nationality^2^ | 1.34 | + | 3.80 | 1.56 | + | 4.77 | 0.91 | + | 2.48 | 1.29 | * | 3.64 |
|  | (0.77) | |  | (0.81) | |  | (0.55) | |  | (0.66) | |  |
| PhD – abroad^3^ | -1.01 |  |  | -1.09 |  |  | -0.71 |  |  | -0.26 |  |  |
|  | (0.70) | |  | (0.71) | |  | (0.60) | |  | (0.67) | |  |
| ¹ reference category is woman  ² reference category is non-UK  ^3^ reference category is PhD from UK-institution  *** p<.001, ** p<.01, *p<.05, +p<.1  *Note: Odds ratios (OR) are only shown for significant estimates* | | | | | | | | | | | | |

Table A2. Linear logistic regression models for High quality data, Reproducing findings, Designing experiments, and High quality science with control for the location of the PhD institution (standard errors are shown between brackets).

Table A3. Linear logistic regression models for Regulatory compliance, Ethics, and Public support (standard errors are shown between brackets).

|  | Regulatory compliance (N=209) | | Ethics  (N=205) | | Public support  (N=202) | |
| --- | --- | --- | --- | --- | --- | --- |
| Constant | 3.69 | ** | 5.21 | *** | 1.64 |  |
|  | (1.21) | | (1.44) | | (0.91) | |
| Gender^1^ | -1.15 |  | 0.07 |  | 0.40 |  |
|  | (0.69) | | (0.71) | | (0.48) | |
| Age | -0.02 |  | -0.06 |  | -0.00 |  |
|  | (0.03) | | (0.03) | | (0.02) | |
| Nationality^2^ | 0.90 |  | 1.12 |  | 0.79 |  |
|  | (0.62) | | (0.79) | | (0.49) | |
| ¹ reference category is woman  ² reference category is non-UK  *** p<.001, ** p<.01, *p<.05  *Note: Odds ratios (OR) are only shown for significant estimates* | | | | | | |
